# Deep learning significantly boosts CRT response prediction using synthetic longitudinal strain data: Training on synthetic data and testing on real patients

**DOI:** 10.1016/j.bj.2024.100803

**Published:** 2024-10-28

**Authors:** Ying-Feng Chang, Kun-Chi Yen, Chun-Li Wang, Sin-You Chen, Jenhui Chen, Pao-Hsien Chu, Chao-Sung Lai

**Affiliations:** a Artificial Intelligence Research Center, Chang Gung University, Taoyuan, Taiwan; b Department of Gastroenterology and Hepatology, New Taipei Municipal Tu Cheng Hospital (Built and Operated by Chang Gung Medical Foundation), New Taipei, Taiwan; c Division of Cardiology, Department of Internal Medicine, Linkou Medical Center, Chang Gung Memorial Hospital, Taoyuan, Taiwan; d Graduate Institute of Clinical Medical Sciences, College of Medicine, Chang Gung University, Taoyuan, Taiwan; e School of Medicine, Chang Gung University, Taoyuan, Taiwan; f Department of Computer Science and Information Engineering, Chang Gung University, Taoyuan, Taiwan; g Division of Breast Surgery and General Surgery, Department of Surgery, Chang Gung Memorial Hospital, Linkou Branch, Taoyuan, Taiwan; h Department of Electronic Engineering, Ming Chi University of Technology, New Taipei, Taiwan; i Institute of Stem Cell and Translational Cancer Research, Chang Gung Memorial Hospital, Taoyuan, Taiwan; j Institute of Pioneer Semiconductor Innovation, Industry Academia Innovation School, National Yang Ming Chiao Tung University, Hsinchu, Taiwan

**Keywords:** Cardiac resynchronization therapy, Deep learning, Treatment response prediction

## Abstract

**Background:**

Recently, as a relatively novel technology, artificial intelligence (especially in the deep learning fields) has received more and more attention from researchers and has successfully been applied to many biomedical domains. Nonetheless, just a few research works use deep learning skills to predict the cardiac resynchronization therapy (CRT)-response of heart failure patients.

**Objective:**

We try to use the deep learning-based technique to construct a model which is used to predict the CRT response of patients with high prediction accuracy, precision, and sensitivity.

**Methods:**

Using two-dimensional echocardiographic strain traces from 131 patients, we pre-processed the data and synthesized 2000 model inputs through the synthetic minority oversampling technique (SMOTE). These inputs trained and optimized deep neural networks (DNN) and one-dimensional convolution neural networks (1D-CNN). Visualization of prediction results was performed using t-distributed stochastic neighbor embedding (t-SNE), and model performance was evaluated using accuracy, precision, sensitivity, F1 score, and specificity. Variable importance was assessed using Shapley additive explanations (SHAP) analysis.

**Results:**

Both the optimal DNN and 1D-CNN models demonstrated exceptional predictive performance, with prediction accuracy, precision, and sensitivity all around 90%. Furthermore, the area under the receiver operating characteristic curve (AUROC) of the optimal 1D-CNN and DNN models achieved 0.8734 and 0.9217, respectively. Crucially, the most significant input variables for both models align well with clinical experience, further corroborating their robustness and applicability in real-world settings.

**Conclusions:**

We believe that both the DL models could be an auxiliary to help in treatment response prediction for doctors because of the excellent prediction performance and the convenience of obtaining input data to predict the CRT response of patients clinically.

## 1 Introduction

Heart failure, a severe cardiovascular disorder, is fundamentally triggered by the extensive damage inflicted on the heart muscle. This often results in a destructive pattern of asynchronous contraction and relaxation of the left and right ventricles, leading to a significant reduction in the heart's pumping capability [1]. This reduction severely impacts the quality of life of affected individuals and necessitates the pursuit of efficient therapeutic options. One such solution that has proven particularly effective is cardiac resynchronization therapy (CRT). CRT has been established as a crucial therapeutic strategy for heart failure patients, especially those exhibiting left ventricular systolic dysfunction and a wide QRS complex [2,3]. This treatment method offers substantial benefits by enabling the heart to contract more synchronously, thus improving its overall pumping function [[4], [5], [6], [7]]. However, while CRT stands as a highly effective solution for many, it isn't universally successful. Alarmingly, approximately 20%–40% of patients exhibit a less-than-favorable response to the therapy [2,[7], [8], [9]]. This situation has driven researchers to design various evaluation strategies to help doctors accurately diagnose a patient belonging to CRT response or CRT non-response. The ultimate aim of these endeavors is to enable doctors to accurately predict a patient's potential response to CRT — whether positive or negative.

Among the numerous CRT response estimation strategies that have been proposed, a couple of them have become the standard practice in the clinical field. For instance, myocardial work and longitudinal strain are routinely utilized to predict a patient's CRT response [2,8,[10], [11], [12], [13]]. Myocardial work, which involves the use of longitudinal strain to measure myocardial deformation and the integration of left ventricular (LV) pressure-strain loops to measure the brachial artery blood pressure, has shown superior predictive performance [8,12]. These measurements offer a comprehensive understanding of the myocardial work performed both globally and at the segment level. To further enhance the accuracy of these predictions, recent advanced studies have combined myocardial work assessments with high-resolution imaging technologies. Examples of these imaging techniques include positron emission tomography (PET) and cardiac magnetic resonance (CMR), which are used to glean more detailed pathophysiological information about the left ventricle [2,7]. This multi-modal approach has yielded impressive results, with prediction accuracies reaching 80% and 85% for PET and CMR, respectively. This significantly outperforms the predictive capabilities of using myocardial work as a standalone measure [2,7]. On the other hand, some investigators have shifted their focus towards the use of biomarkers for predicting CRT response. These include traditional heart failure biomarkers (e.g., NT-pro-BNP, BNP, and hsTnT) as well as inflammation-related biomarkers (e.g., hsCRP and complement C3a) [6,9,14,15]. While a definitive, highly predictive biomarker for CRT response remains elusive, the possibility of combining multiple biomarkers into a "signature panel" has sparked considerable excitement and hope in the scientific community [14].

Recently, deep learning technology has garnered considerable attention from researchers across various fields, including the medical industry. It has been successfully implemented in various biomedical applications [[16], [17], [18], [19], [20], [21], [22], [23]]. Although many machine learning techniques have been utilized to predict a patient's response to cardiac resynchronization therapy (CRT), to the best of our knowledge, only a few studies have used deep learning methods to enhance CRT response prediction [[24], [25], [26], [27], [28], [29], [30], [31], [32]]. This is largely due to the fact that, while mature deep learning-based computer vision models are widely available and convenient to access, relying solely on these models for predicting CRT response is inadequate. To address this issue, we must customize specific prediction models, given that they typically require medical data inputs rather than merely image-based inputs, differentiating them from conventional computer vision applications.

In this study, we propose a simple approach to this challenge by introducing two models designed to predict CRT response in patients — a deep neural networks (DNN)-based model and a one-dimensional convolution neural network (1D–CNN)–based model. These models have demonstrated impressive results, with prediction accuracy, precision, and sensitivity all exceeding 90%. Moreover, these models only require longitudinal strain traces as the input, which is both straightforward and convenient to obtain. Therefore, we are confident that this approach carries high potential for real-world application, given its impressive prediction accuracy and practicality in a clinical setting.

## 2 Methods

### 2.1 Patients and echocardiography

In this study, 131 patients (CRT response: 91 patients and CRT non-response: 40 patients) who were treated with the CRT in the Division of Cardiology, Chang Gung Memorial Hospital (CGMH) were collected from 2011/12 to 2020/07 (8 years and 8 months) and the baseline characteristics of patients were shown in [Table 1]. The study protocol complied with the Declaration of Helsinki and was approved by the Institutional Review Board of CGMH (the certificate no. 202002509B0). For the 131 collected patients in this study, the two-dimensional (2D) echocardiography and left ventricular end-systolic volume (LVESV) measurement were performed twice when the patient before CRT implantation and after six months as the baseline and follow-up, respectively. The LV volumes were measured using the biplane Simpson's method and the CRT response was defined as: “compared with the baseline, the LVESV decreased by over 15% after six months of CRT”. The two-dimensional echocardiography with 50–80 Hz frame rates were obtained from the apical 4-chamber (4C), 3-chamber (2C), and 2-chamber (2C) views to analyze the speckle-tracking longitudinal strain. Subsequently, the software, i.e. EchoPAC (General Electric Vingmed Ultrasound), was used to trace the endocardial border in each view, and then automatically tracked the image speckle to produce six longitudinal time-series strain traces of each apical view as the raw data.

**Table 1:** Baseline characteristics.

	Entire population (n = 131)	CRT response (n = 91)	CRT non-response (n = 40)	P value
Age, years	69.0 ± 12.0	70.0 ± 11.4	66.7 ± 13.1	0.153
Male (%)	71 (54.2)	45 (49.5)	26 (65.0%)	0.100
NYHA class (II/III/IV)	44/81/6	33/55/3	11/26/3	0.010
Diabetes mellitus (%)	59 (45)	42 (46.2)	17 (42.5)	0.699
Hypertension (%)	86 (65.6)	64 (70.3)	22 (55)	0.089
Ischemic cardiomyopathy (%)	49 (37.4)	23 (25.3)	26 (65.0)	<0.001
SBP, mmHg	120.4 ± 19.5	122.5 ± 19.8	115.6 ± 18.1	0.060
DBP, mmHg	70.8 ± 11.8	71.5 ± 12.3	69.3 ± 10.7	0.327
Body mass index, kg/m^2^	24.1 ± 3.6	24.3 ± 3.8	23.6 ± 3.0	0.252
β-blockers	116 (88.5)	79 (92.9)	33 (84.6)	0.145
ACEi/ARB	114 (87.0)	82 (90.1)	32 (80.0)	0.113
MRA	71 (54.2)	53 (58.2)	18 (45.0)	0.161
Baseline QRS duration, ms	162.1 ± 19.2	162.5 ± 19.3	161.1 ± 19.1	0.696
QRS duration shortening∗, ms	11.3 ± 22.0	16.2 ± 20.2	−0.1 ± 22.3	<0.001
eGFR, mL/min/1.73m^2^	63.1 ± 30.8	65.4 ± 31.4	57.8 ± 29.2	0.200
LVEDV, mL	172.4 ± 68.1	160.1 ± 60.4	199.6 ± 76.5	0.002
LVESV, mL	133.9 ± 63.1	122.7 ± 55.4	158.9 ± 71.9	0.002
LVEF, (%)	24.5 ± 7.6	25.5 ± 6.8	22.2 ± 8.6	0.019

Abbreviation: NYHA: New York Heart Association; SBP: systolic blood pressure; DBP: diastolic blood pressure; ACEi: angiotensin-converting enzyme inhibitor; ARB: angiotensin receptor blocker; MRA: mineralocorticoid receptor antagonist; QRS duration shortening∗: baseline QRS duration – post-CRT QRS duration(ms); eGFR: estimated glomerular filtration rate; LVESV: left ventricular end-diastolic volume; LVESV: left ventricular end-systolic volume. LVEF: left ventricular ejection fraction.

### 2.2 Data pre-processing and the model input

Due to the frequency of heartbeat exhibiting a huge difference between patients, the length of the time-series strain traces which were used as the model input in this study was so different between patients as well. Thus, we first normalized the length of the time-series strain traces for all patients through the data pre-processing procedure. Experimentally, the six longitudinal time-series strain traces of each apical view (4C, 3C, and 2C views) were used as the raw data to transform into the corresponding heartbeat-series data through the data pre-processing to normalized the length of the input. The data pre-processing was described as follows.

1. All the data points of the time-series strain traces were first divided by the period of heartbeat to transform into its corresponding heartbeat-series strain traces.
2. In order to normalize the data length, we set a fixed sample rate of heartbeat for all the 131 patients and the lack of data points of the heartbeat-series strain traces were produced by the interpolation method. Where the sample rate of heartbeat refers to the number of sampled times per heartbeat.

**Fig. 1:**
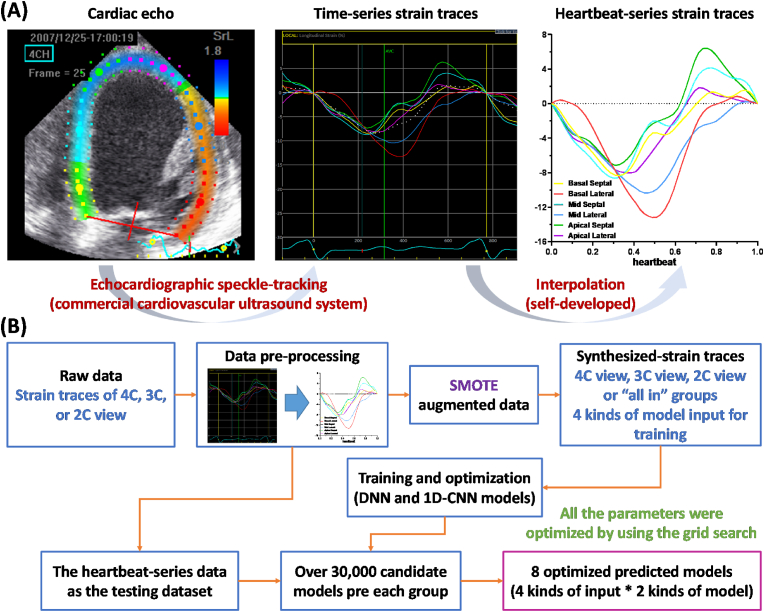
(A) Diagram of data pre-processing. (B) The constructed procedure of the CRT response prediction model.

Thus, the whole data pre-processing [see Fig. 1A] was to transform the raw data (six time-series strain traces) into the six heartbeat-series strain traces in which the length was the same between patients and used as the input in this study.

### 2.3 The construction procedure of the CRT response prediction models

The construction procedure of the proposed CRT response prediction model is illustrated in [Fig. 1B]. First, we transformed the time-series strain traces (raw data) of the 131 patients into their corresponding heartbeat-series strain traces. Subsequently, we augmented the heartbeat-series strain traces for each apical view [4C, 3C, and 2C] by applying the synthetic minority oversampling technique (SMOTE). Specifically, for each patient, the six heartbeat-series strain traces of each apical view were treated as one set. Using SMOTE, we individually augmented the 91 sets of heartbeat-series strain traces from CRT response patients to 1365 synthesized-strain sets for each apical view [4C, 3C, or 2C]. Similarly, we individually augmented the 40 sets of heartbeat-series strain traces from CRT non-response patients to 635 synthesized-strain sets for each apical view. The 2000 sets of synthesized-strain traces of each apical view were used to train and validate their respective DNN and 1D-CNN models. Since our training data was synthesized from real heartbeat-series strain traces via SMOTE, we applied a 5-fold cross-validation approach to mitigate the risk of overfitting during the training phase. In addition, besides individually using the synthesized-strain traces of 4C, 3C, or 2C views as the model input during the training step, we try to combine all the synthesized-strain traces of 4C, 3C, and 2C views together, i.e. “all in” group, as the model input as well, to test such input whether reach to a better prediction performance than only using the synthesized-strain traces of single apical view as the model input. Finally, the heartbeat-series strain traces of the 131 patients were used to test the selected prediction models from all candidate models of a grid search method just mentioned below.

**Fig. 2:**
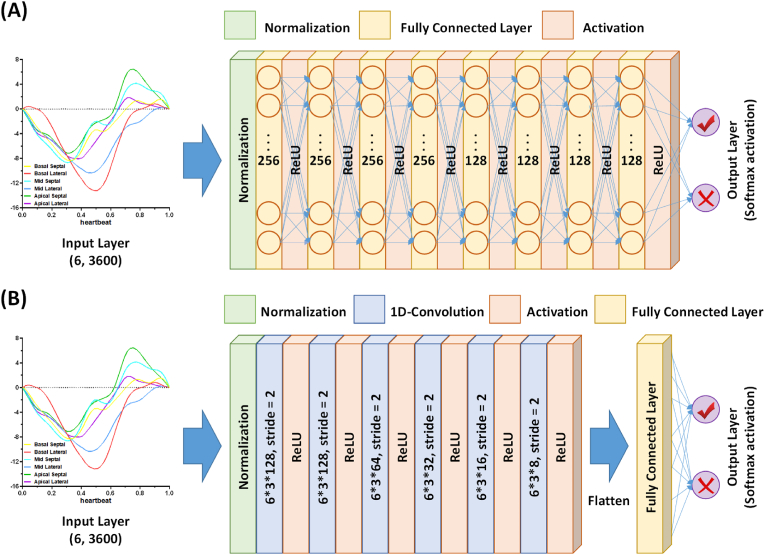
(A) Diagram of the optimal DNN model. (b) Diagram of the optimal 1D-CNN model.

In the training and optimization process, all the hyperparameters and architectures of the DNN and 1D-CNN models were optimized by using the grid search technique. The preliminary options for each parameter were: (a) training batch size: 16, 32, 64, and 128. (b) dropout: 0, 0.1, and 0.3. (c) learning rate: 0.001, 0.003, and 0.01. (d) epoch: 125. (e) the number of hidden layers for the both models: 3, 4, 5, 6, 7, 8, and 9. (f) the number of the cells for each hidden layer: 8, 16, 32, 64, 128, and 256. The loss functions for all models were mean absolute error (MAE). The structure of the optimal DNN and 1D-CNN models as shown in [Fig. 2] and all models were trained on Leadtek WinFast HS5800 with 12 NVIDIA V100 GPUs (16 GB graphical memories). In addition, both the DNN and 1D-CNN models were also optimized the different sample rates (1/36, 1/72, 1/144, 1/360, 1/720, 1/1800, 1/3600) to aim the best prediction performance.

### 2.4 Visualization of prediction results

In this study, we used the t-distributed stochastic neighbor embedding (t-SNE) technique to visualize the prediction results of different training epochs. Basically, t-SNE uses conditional probability and Gaussian distribution to define the similarity between sample points in high and low dimensions and uses Kullback-Leibler divergence, a.k.a. relative entropy, to measure the similarity between the sum of two conditional probability distributions [33]. In addition, the t-distribution was used in this method to define the probability distribution at low dimensions to mitigate the crowding problem caused by the curse of dimensionality [33]. Due to the above-mentioned, t-SNE exhibits an excellent ability to preserve local structure during dimensionality reduction.

### 2.5 Variable importance of the optimized models

In order to understand the importance of the input variables of each sub-model, we try to use the Shapley additive explanations (SHAP) analysis to describe the variable importance in this study. SHAP analysis which is a cooperative game theory-based approach usually uses to show the importance, a.k.a. contribution, of every input variable on the prediction of the model to increase the interpretability and transparency of the deep learning models.

### 2.6 Evaluation indexes

The five usual evaluation indexes including accuracy, precision (also called positive predictive value, PPV), sensitivity (also known as recall), F1 score, and specificity were used to review the prediction performance of the proposed deep learning models. These evaluation indexes respective definitions are listed below:

Accuracy = (TP + TN)/(TP + FP + TN + FN)

Precision (Positive Predictive Value, PPV) = TP/(TP + FP)

Sensitivity (Recall) = TP/(TP + FN)

F1 score = 2 ✕ Sensitivity ✕ Precision/(Sensitivity + Precision)

Specificity = TN/(TN + FP)where TP is the number of true positives, TN is the number of true negatives, FP is the number of false positives, and FN is the number of false negatives.

## 3 Results

### 3.1 Optimization of the sample rates

**Fig. 3:**
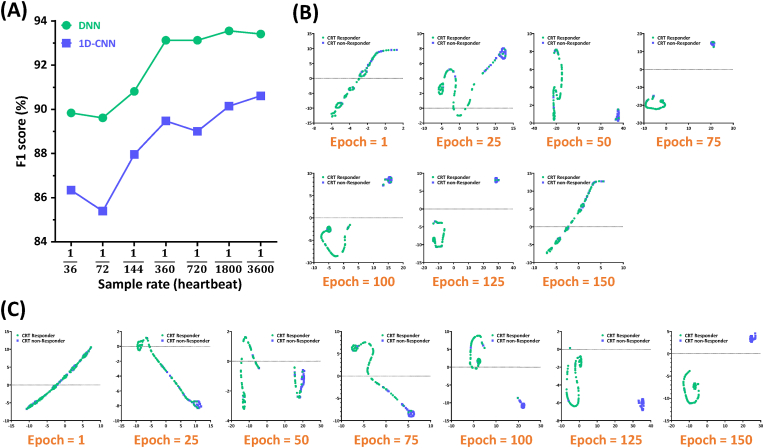
(A) The prediction performance of the optimized 4CH-DNN and 4CH-1D-CNN models with different sample rates. Visualization of the prediction results of the (B) optimal DNN model and (C) optimal 1D-CNN model by t-SNE technique.

In this study, the six longitudinal time-series strain traces for each apical view (4C, 3C, or 2C view) were used as the raw data and were transformed into the corresponding heartbeat-series data by the data pre-processing procedure (see “Data pre-processing and the model input” section). The sample rate of the heartbeat which is a key factor during the data pre-processing procedure refers to the number of sampled times per heartbeat. For instance, if we set the sample rate as “1/10” and “1/1000”, then we use 10 and 1000 data points to describe one heartbeat in heartbeat-series data, respectively. It is easy to imagine that there are more data points to describe the heartbeat, the more resembling between the raw data and heartbeat-series data. Thus, the sample rate of the heartbeat strongly dominates the similarity which significantly influences the prediction performance of the proposed prediction models, between the raw data and heartbeat-series data, because the heartbeat-series data is the input of the proposed prediction models. It is totally impracticable to expect the proposed prediction models to show an outstanding prediction performance in which the input heartbeat-series data is not similar to the raw data. In order to aim for the best prediction performance, we optimize the sample rate of the heartbeat and used the F1 score as the index to evaluate the prediction performance of the models as shown in [Fig. 3A]. In this figure, although the F1 score of the DNN model rises rapidly in the beginning, the F1 score goes to a steady level around 94 when the sample rate is lower than “1/360”. While the F1 scores of the 1D-CNN model also exhibit a larger increase at the beginning than at the sample rate lower than “1/360”. Since the F1 score of the both models are higher than 90 when the sample rate is equal to “1/3600”, thus we select “1/3600” as the optimized sample rate for both models.

### 3.2 Prediction performance of the proposed deep learning models

In this study, all the candidate models from the grid search of hyperparameters and architectures were respectively and independently trained using the SMOTE-synthesized data including 4C view, 3C view, 2C view and the all in groups (mixing the aforementioned three views). During the training, the prediction performance of all the candidate models was reviewed by the area under the receiver operating characteristic curve (AUROC) to identify the optimal prediction models. The AUROC values of the optimal DNN and 1D-CNN models for the 5-fold cross-validation are shown in [Table 2], where the models using the 4C view or all in groups as model input exhibit better prediction performance compared to those using the 3C view or 2C view groups.

**Table 2:** The AUROC values of the optimal DNN and 1D-CNN models in 5-fold cross-validation using the SMOTE-synthesized data.

Apical view of strain traces	AUROC of DNN model (%)	AUROC of 1D-CNN model (%)
4-chamber view	92.56 ± 4.90	92.11 ± 1.61
3-chamber view	82.60 ± 5.46	82.90 ± 5.70
2-chamber view	85.10 ± 4.93	85.20 ± 5.67
All in	90.80 ± 15.91	93.60 ± 3.10

**Fig. 4:**
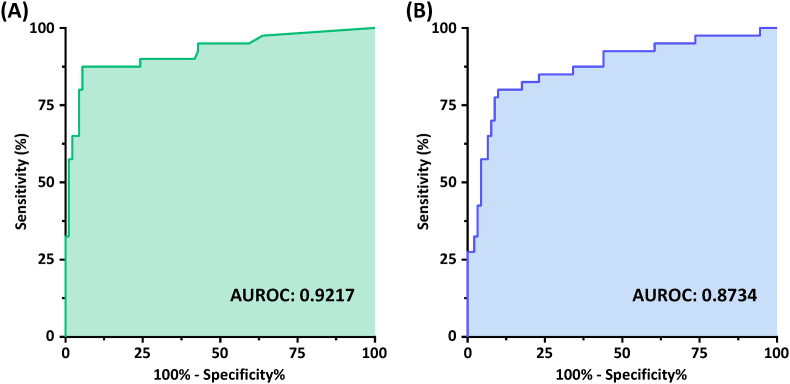
(A) The AUROC of the optimized DNN model. (B) The AUROC of the optimized 1D-CNN model.

[Table 3] shows the prediction performances in the 131 patients of the optimal DNN and 1D-CNN models of the four input groups [4C, 3C, 2C, and all in]. Initially, we expected that the all in group will show the best prediction performance because it integrates all the features from 4C, 3C, and 2C views as the input. However, the overall results of the all in group were unexpectedly not the best for the DNN model and 1D-CNN model, the prediction performance was actually more like the average of the 4C, 3C, and 2C views. We believe that using the stacking strategy of ensemble learning should easily solve this issue if necessary. Experimentally, the 4C view group exhibited the winning a championship with an excellent record on four evaluation indexes (accuracy, precision, F1 score, and specificity) except for the sensitivity (recall), and moreover, its sensitivity was still higher than 90%. In addition, the DNN-based prediction model of the 4C view group is the only one that exhibits a prediction accuracy of over 90% in this study and this result is one of the best performances for the prediction of CRT response in literature [2,7,8,10,14,24,30,34]. Although the 2C view group showed the best performance around 95% on the sensitivity (recall) both for the DNN model and 1D-CNN model, the specificity of them were both less than 60%. [Fig. 4] displays the ROC curves of both 4C view optimal models, and their AUROCs are 0.9217 and 0.8734 for the optimal DNN model and 1D-CNN model, respectively. Thus, based on our experimental results, using the heartbeat-series data obtained from the 4C view as the input for the optimized DNN and 1D-CNN models could be the best choice to predict the CRT response of patients.

**Table 3:** Performance metrics of optimal prediction models for various apical views in the 131 patients.

Performance metrics of the optimal DNN prediction models for different apical views					
Apical view of strain traces^a^	Accuracy (%)	Precision (%)	Sensitivity (%)	F1-score (%)	Specificity (%)
4-chamber view	91.60	93.47	94.51	93.99	85.00%
3-chamber view	87.79	88.66	94.51	91.49	72.50%
2-chamber view	83.21	83.50	94.51	88.66	57.50%
All in	86.26	87.63	93.41	90.43	70.00%

Performance metrics of the optimal 1D-CNN prediction models for different apical views					
Apical view of strain traces^a^	Accuracy (%)	Precision (%)	Sensitivity (%)	F1-score (%)	Specificity (%)
4-chamber view	87.02	91.11	90.11	90.61	80.00%
3-chamber view	79.39	82.00	90.11	85.86	55.00%
2-chamber view	79.39	79.09	95.60	86.57	42.50%
All in	81.68	87.64	85.71	86.67	72.50%

a Six strain traces encompass the basal, mid, and apical segments of the septal and lateral walls in the 4-chamber view, the anteroseptal and posterior walls in the 3-chamber view, and the inferior and anterior walls in the 2-chamber view.

### 3.3 Visualization of the best prediction results

In this study, the t-SNE technique was used to visualize the best prediction results as shown in [Fig. 3]. T-SNE has been often used to transform the data in a high-dimensional feature space into a low-dimensional feature space, which can be used to visualize each different cluster in a two-dimensional space and therefore make the model learning easier to be checked layer by layer [33,[35], [36], [37]]. [Fig. 3B] and C respectively show the visualization of the layered prediction results of the optimized DNN and optimized 1D-CNN models which use the 4C view based strain traces as the input. In the beginning, the outputs of both optimized models do not display two clear clusters, they were all un-differentiated. Although the model inevitably misjudges a few patients, both the figures present two more and more clear clusters (green: CRT responder & blue: CRT non-responder) in larger epochs (≥50 epochs). In addition, for 150 epochs, the clusters of CRT responder and CRT non-responder which were predicted by the optimized DNN model were back to un-differentiated, thus we used 125 epochs during training and optimizations to reach the best prediction performance.

### 3.4 Variable importance of the optimized models

**Fig. 5:**
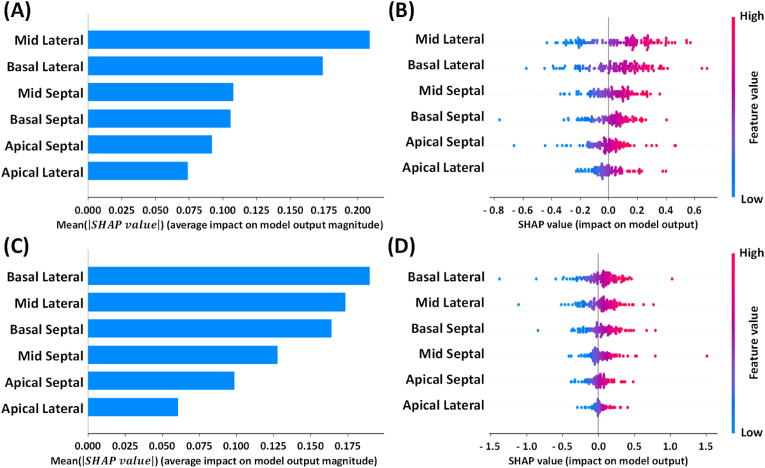
(A) The global effect (importance) of input variable for the optimized DNN model. (B) The importance of the input variables of the optimized DNN model for the whole dataset. (C) The global effect (importance) of input variable for the optimized 1D-CNN model. (D) The importance of the input variables of the optimized 1D-CNN model for the whole dataset.

In order to understand the importance of the model input of the optimal DNN and 1D-CNN models for 4C view group, we try to use the Shapley additive explanations (SHAP) analysis to describe the input importance in this study. SHAP analysis which is a cooperative game theory-based approach usually uses to show the importance, a.k.a. contribution, of every input variable on the prediction of the model to increase the interpretability and transparency of the deep learning models. [Fig. 5A and C] shows the global effect (importance) of input variables through the absolute SHAP values of the optimal models for 4C view data. The results show that the apical strain traces always exhibit a less effect on both models than the strain traces of other parts and the strain traces of mid and basal lateral significantly affect both models. In addition, the importance of the input variables for the whole dataset exhibit the same results as shown in [Fig. 5B and D].

Since both the apical strain traces show relatively less importance to the prediction of CRT response, we try to re-train and re-optimize the DNN and 1D-CNN models by the synthesized-strain sets without the apical strain traces and the results of testing the 131 patients are shown in [Table 4]. It clearly presents that using the four heartbeat-series strain traces, i.e. basal lateral, mid lateral, basal septal, and mid septal, as the model input, results in a better prediction performance only for the 1D-CNN model. The overall prediction performance decreases slightly for the DNN re-optimized model. Thus, based on our experimental results, the DNN model is need to use all the six heartbeat-series strain traces as the model input to achieve the best prediction performance, while the 1D-CNN model only needs to use the four heartbeat-series strain traces as the model input to realize this.

**Table 4:** Predictive performance of the optimal models using four strain traces^a^ and six strain traces^b^ from the 4-chamber view as input.

Optimized model	Accuracy (%)	Precision (%)	Sensitivity (%)	F1-score (%)	Specificity (%)
DNN (Six strain traces)	91.60	93.47	94.51	93.99	85.00
DNN (Four strain traces)	90.08	91.49	94.51	92.97	80.00
1D-CNN (Six strain traces)	87.02	91.11	90.11	90.61	80.00
1D-CNN (Four strain traces)	90.08	92.39	93.41	92.90	82.50

a Four strain traces were obtained from the basal-lateral, mid-lateral, basal-septal, and mid-septal segments.

b Six strain traces were derived from the basal-lateral, mid-lateral, apical-lateral, basal-septal, mid-septal, and apical-septal segments.

## 4 Discussion

### 4.1 Comparison between DNN and 1D-CNN

In our study, although the DNN model performed better overall, it is essential to understand the potential advantages of both the 1D-CNN and DNN models and the reasons for the observed performance differences. 1D-CNNs typically offer computational efficiency due to their convolutional layers, which can automatically learn local features and perform parallel computations, making them more suitable for large datasets. They are particularly effective at capturing local dependencies and patterns in data, and their parameter-sharing mechanisms usually require fewer parameters compared to DNNs, which helps reduce the risk of overfitting, especially when the amount of data is limited. On the other hand, DNNs are generally better at learning and capturing global patterns in data, which is beneficial for complex tasks where understanding the overall structure and relationships within the data is crucial. DNNs also typically have a higher capacity for learning complex functions due to their deeper architecture and larger number of parameters, making them advantageous for datasets with intricate and high-dimensional patterns. While both models have their respective advantages, our current results suggest that the data characteristics in our study are more suitable for DNNs. This suggests that the particular patterns and relationships in our dataset are better learned by DNNs. Moreover, the performance of both models can be significantly influenced by hyperparameter settings. Different configurations may lead to varying results, and the settings used in our study might have favored the DNN model.

Comparison of the prediction performance of CRT response between current methods and the proposed models.

As it stands, myriad research groups have conceptualized and promulgated a range of strategies to estimate the response to cardiac resynchronization therapy (CRT), notable among which involve the application of myocardial work and biomarkers to predict CRT response. [Table 5] provides a comparison of the CRT response prediction performance between a selection of recent studies employing these stated methods and the models devised within the framework of our study. Though some investigations utilizing biomarkers to predict patient CRT response yield encouraging results, these are often predicated on the need for expanded sample sizes to bolster broader credibility [9,12,14,15]. Prediction methods premised on myocardial work demonstrate commendable performance, yet to attain these remarkable outcomes necessitates a specific cardiovascular ultrasound system in concert with advanced imaging technologies, such as positron emission tomography (PET) or cardiac magnetic resonance (CMR) [2,7,8]. However, the requisition of such costly and sophisticated imaging apparatus could conceivably constrain the development of this prediction method. Recently, various teams have attempted to harness diverse clinical data — encompassing medical, laboratory, clinical, ECG, and echocardiography records — as input features for conventional machine learning techniques, with the aim to predict patient CRT response [[25], [26], [27], [28], [29], [30]]. Albeit the accuracy of these approaches ranges approximately between 68% and 82% (refer to Table 6), indicating considerable scope for improvement. A handful of studies have sought to deploy deep learning techniques, extracting features from cardiac imaging data and utilizing them in tandem with clinical data, aspiring to achieve superior prediction performance [24,31]. Insight derived from [Table 5, Table 6] reveals that current avant-garde techniques for predicting CRT response are intrinsically reliant on advanced, costly, precise, or specialized imaging systems. In stark contrast, the prediction models introduced in our study exhibit competitive performance, with accuracy and sensitivity exceeding 90%. Moreover, they necessitate solely the strain traces from the 4C view of a cardiovascular ultrasound system for model input. This input eschews the need for additional data from exorbitant imaging systems and can be provisioned by any commercial cardiovascular ultrasound system, thus significantly enhancing practical convenience.

**Table 5:** Prediction performance of CRT response between previous methods and the proposed models.

Prediction methods	Accuracy (%)	Sensitivity (%)	Specificity (%)	Number of patients	Reference
biomarker	84.91^a^	93.5	71.4	53	Clin. Res. Cardiol. 2021 [6]
biomarker	84.21^a^	83.3	90.0	38	ESC Heart fail. 2021 [15]
myocardial work	85^a^	79	82	100	Quant. Imaging Med. Surg. 2021 [8]
myocardial work + CMR	85	86	84	200	Eur. Heart J. 2020 [2]
myocardial work + PET	90	80	83	83	Eur. J. Nucl. Med. Mol. Imaging 2021 [7]
Four strain traces from the 4C view using deep learning (1D-CNN)	90.08	93.41	82.5	131	This study
Six strain traces from the 4C view using deep learning (DNN)	91.60	94.51	85	131	This study

CMR: cardiac magnetic resonance.

PET: positron emission tomography.

a The accuracy was not provided in the paper and was estimated through the sensitivity, specificity, and the number of patients by the authors.

**Table 6:** Comparison of the prediction performance of CRT response between ML methods and the proposed models.

Prediction methods (features)	Accuracy (%)	Sensitivity (%)	Specificity (%)	Number of patients	Reference
naive bayes classifier (clinical data)	68	46	83	470	Circ.-arrhythmia Electrophysiol. 2019 [26]
LR, EN, k-NN, NN, RF, SVM, XGBoost, Ridge, and Lasso (clinical data)	69–74	54–72	66–91	752	Int. J. Cardiol. 2021 [30]
LR, SVM, RF, LDA (clinical data and simulations of the response to biventricular)	82	85	78	57	Front. Physiol. 2021 [28]
DL algorithm (clinical data and echo features)	91.85^a^	89.53^a^	94.19^a^	209	Biocybern. Biomed. Eng. 2021 [24]
DL algorithm (2D CMR and 2D echocardiography images)	77.38	83.33	71.43	50	Med. Image Anal. 2022 [31]
Four strain traces from the 4C view using deep learning (1D-CNN)	90.08	93.41	82.5	131	This study
Six strain traces from the 4C view using deep learning (DNN)	91.60	94.51	85	131	This study

Abbreviations: ML: machine learning; DL: deep learning; LR: logistic regression; EN: elastic network; k-NN: k-nearest neighbor; NN: neural network; RF: random forest; SVM: support vector machine; LDA: linear discriminant analysis.

a The result including synthetic samples.

## 5 Conclusions

In this paper, we proposed two accurate and simple DL models to predict the CRT response of patients. We first normalized the length of the time-series strain traces for all patients through the data pre-processing procedure to heartbeat-series strain traces. Then, we individually augmented the heartbeat-series strain traces of the patients to 2000 synthesized-strain traces by using the SMOTE for each apical view (4C, 3C, and 2C view). Finally, the DL models were trained using the synthesized data and tested by clinical data. Both the DL models exhibit an outstanding prediction performance in which the prediction accuracy, precision, and sensitivity are over 90%. In addition, compared to the state of art approaches, both the proposed models show better prediction accuracy. We believe that both the DL models could be an auxiliary to help in treatment response prediction for doctors because of the excellent prediction performance and the convenience of obtaining input data to predict the CRT response of patients clinically.

## Declaration of generative AI and AI-assisted technologies in the writing process

During the preparation of this work, the authors used GPT-4o in order to edit the English. After using this tool, the authors reviewed and edited the content as needed and take full responsibility for the content of the publication.

## Declaration of competing interest

The authors declare no competing financial interest.

## References

[1] Mann DL, Bristow MR. (2005). Mechanisms and models in heart failure: the biomechanical model and beyond. Circulation.

[2] Aalen JM, Donal E, Larsen CK, Duchenne J, Lederlin M, Cvijic M (2020). Imaging predictors of response to cardiac resynchronization therapy: left ventricular work asymmetry by echocardiography and septal viability by cardiac magnetic resonance. Eur Heart J.

[3] Alpendurada F, Guha K, Sharma R, Ismail TF, Clifford A, Banya W (2011). Right ventricular dysfunction is a predictor of non-response and clinical outcome following cardiac resynchronization therapy. J Cardiovasc Magn Reson.

[4] Feeny AK, Rickard J, Trulock KM, Patel D, Toro S, Moennich LA (2019). Machine learning of 12-lead QRS waveform patterns to identify cardiac resynchronization therapy patients with differential outcomes. Circulation.

[5] Rath B, Willy K, Wolfes J, Ellermann C, Reinke F, Kobe J (2021). Predictors of response to cardiac resynchronization therapy in patients with chronic right ventricular pacing. Clin Res Cardiol.

[6] Sultan A, Wormann J, Luker J, van der Bruck JH, Plenge T, Rudolph V (2021). Significance of myeloperoxidase plasma levels as a predictor for cardiac resynchronization therapy response. Clin Res Cardiol.

[7] Degtiarova G, Claus P, Duchenne J, Bogaert J, Nuyts J, Voros G (2021). Left ventricular regional glucose metabolism in combination with septal scar extent identifies CRT responders. Eur J Nucl Med Mol Imag.

[8] Zhu MR, Wang YN, Cheng YF, Su YA, Chen HY, Shu XH. (2021). The value of non-invasive myocardial work indices derived from left ventricular pressure-strain loops in predicting the response to cardiac resynchronization therapy. Quant Imag Med Surg.

[9] Massoullie G, Sabin V, Ploux S, Rossignol P, Mulliez A, Jean F (2019). Low fibrosis biomarker levels predict cardiac resynchronization therapy response. Sci Rep.

[10] Risum N, Tayal B, Hansen TF, Bruun NE, Jensen MT, Lauridsen TK (2015). Identification of typical left bundle branch block contraction by strain echocardiography is additive to electrocardiography in prediction of long-term outcome after cardiac resynchronization therapy. J Am Coll Cardiol.

[11] Leenders GE, Lumens J, Cramer MJ, De Boeck BWL, Doevendans PA, Delhaas T (2012). Septal deformation patterns delineate mechanical dyssynchrony and regional differences in contractility analysis of patient data using a computer model. Circulation-Heart Failure.

[12] Galli E, Oger E, Aalen JM, Duchenne J, Larsen CK, Sade E (2021). Left atrial strain is a predictor of left ventricular systolic and diastolic reverse remodelling in CRT candidates. Eur Heart J Cardiovascu Imag.

[13] Hubert A, Gallard A, Le Rolle V, Smiseth OA, Leclercq C, Voigt JU (2021). Left ventricular strain for predicting the response to cardiac resynchronization therapy: two methods for one question. Eur Heart J Cardiovascu Imag.

[14] Heggermont W, Auricchio A, Vanderheyden M. (2019). Biomarkers to predict the response to cardiac resynchronization therapy. Europace.

[15] Yang SW, Hu YR, Zhao JH, Jing R, Wang J, Gu M (2021). Comprehensive plasma metabolites profiling reveals phosphatidylcholine species as potential predictors for cardiac resynchronization therapy response. ESC Heart Failure.

[16] Jamshidi MB, Lalbakhsh A, Talla J, Peroutka Z, Hadjilooei F, Lalbakhsh P (2020). Artificial intelligence and COVID-19: deep learning approaches for diagnosis and treatment. IEEE Access.

[17] Song Y, Zheng SJ, Li L, Zhang X, Zhang XD, Huang ZW (2021). Deep learning enables accurate diagnosis of novel coronavirus (COVID-19) with CT images. IEEE ACM Trans Comput Biol Bioinf.

[18] Oh SL, Hagiwara Y, Raghavendra U, Yuvaraj R, Arunkumar N, Murugappan M (2020). A deep learning approach for Parkinson’s disease diagnosis from EEG signals. Neural Comput Appl.

[19] Tajbakhsh N, Jeyaseelan L, Li Q, Chiang J, Wu ZH, Ding XW (2020). Embracing imperfect datasets: a review of deep learning solutions for medical image segmentation. Med Image Anal.

[20] Aggarwal R, Sounderajah V, Martin G, Ting DSW, Karthikesalingam A, King D (2021). Diagnostic accuracy of deep learning in medical imaging: a systematic review and meta-analysis. NPJ Digit Med..

[21] Esteva A, Chou K, Yeung S, Naik N, Madani A, Mottaghi A (2021). Deep learning-enabled medical computer vision. NPJ Digit Med..

[22] Castiglioni I, Rundo L, Codari M, Di Leo G, Salvatore C, Interlenghi M (2021). AI applications to medical images: from machine learning to deep learning. Physica Medica-Eur. J Med Phys.

[23] Chang YF, Chen SY, Lee CC, Chen JH, Lai CS. (2022). Easy and rapid approach to obtaining the binding affinity of biomolecular interactions based on the deep learning boost. Anal Chem.

[24] Nejadeh M, Bayat P, Kheirkhah J, Moladoust H. (2021). Predicting the response to cardiac resynchronization therapy (CRT) using the deep learning approach. Biocybern Biomed Eng.

[25] Feeny AK, Rickard J, Trulock KM, Patel D, Toro S, Varma N (2019). Machine learning prediction of cardiac resynchronization therapy response using 12-lead QRS waveform changes after biventricular pacing. Circulation.

[26] Feeny AK, Rickard J, Patel D, Toro S, Trulock KM, Park CJ (2019). Machine learning prediction of response to cardiac resynchronization therapy improvement versus current guidelines. Circula Arrhyth Electrophysiol.

[27] Cikes M, Sanchez-Martinez S, Claggett B, Duchateau N, Piella G, Butakoff C (2019). Machine learning-based phenogrouping in heart failure to identify responders to cardiac resynchronization therapy. Eur J Heart Fail.

[28] Khamzin S, Dokuchaev A, Bazhutina A, Chumarnaya T, Zubarev S, Lyubimtseva T (2021). Machine learning prediction of cardiac resynchronisation therapy response from combination of clinical and model-driven data. Front Physiol.

[29] Feeny A, Rickard J, Patel D, Toro S, Trulock K, Park C (2019). Machine learning prediction of echocardiographic response and survival in cardiac resynchronization therapy. J Am Coll Cardiol.

[30] Liang YX, Ding RF, Wang JF, Gong X, Yu ZQ, Pan L (2021). Prediction of response after cardiac resynchronization therapy with machine learning. Int J Cardiol.

[31] Puyol-Anton E, Sidhu BS, Gould J, Porter B, Elliott MK, Mehta V (2022). A multimodal deep learning model for cardiac resynchronisation therapy response prediction. Med Image Anal.

[32] Wouters PC, van de Leur RR, Vessies MB, van Stipdonk AMW, Ghossein MA, Hassink RJ (2023). Electrocardiogram-based deep learning improves outcome prediction following cardiac resynchronization therapy. Eur Heart J.

[33] Cheng YC, Wang XL, Xia YS (2021). Supervised t-distributed stochastic neighbor embedding for data visualization and classification. Inf J Comput.

[34] Ghani A, Delnoy PP, Ottervanger JP, Ramdat Misier AR, Smit JJ, Adiyaman A (2016). Are changes in the extent of left ventricular dyssynchrony as assessed by speckle tracking associated with response to cardiac resynchronization therapy?. Int J Cardiovasc Imag.

[35] Hammad M, Hewahi N, Elmedany W. (2022). MMM-RF: a novel high accuracy multinomial mixture model for network intrusion detection systems. Comput Secur.

[36] Xu JL, Cui LY, Zhuang JJ, Meng YJ, Bing PP, He BS (2022). Evaluating the performance of dropout imputation and clustering methods for single-cell RNA sequencing data. Comput Biol Med.

[37] Prudente VHR, Skakun S, Oldoni LV, Xaud HAM, Xaud MR, Adami M (2022). Multisensor approach to land use and land cover mapping in Brazilian Amazon. ISPRS J Photogrammetry Remote Sens.

